# Pre-vaccine serotype composition within a lineage signposts its serotype replacement – a carriage study over 7 years following pneumococcal conjugate vaccine use in the UK

**DOI:** 10.1099/mgen.0.000119

**Published:** 2017-06-09

**Authors:** Rebecca A. Gladstone, Vanessa Devine, Jessica Jones, David Cleary, Johanna M. Jefferies, Stephen D. Bentley, Saul N. Faust, Stuart C. Clarke

**Affiliations:** ^1^​ Infection Genomics, Wellcome Trust Sanger Institute, Wellcome Trust Genome Campus, Hinxton, Cambridge, CB10 1SA, UK; ^2^​ Faculty of Medicine and Institute for Life Sciences, University of Southampton, UK; ^3^​ Southampton NIHR Respiratory Biomedical Research Unit, University Hospital Southampton Foundation NHS Trust, Southampton, UK; ^4^​ NIHR Wellcome Trust Clinical Research, Manchester Royal Infirmary, Grafton St, Manchester M13 9WL, UK

**Keywords:** *Streptococcus pneumoniae*, pneumococcal, vaccines, PCV, serotype, replacement, whole genome sequencing

## Abstract

Serotype replacement has been reported in carriage and disease after pneumococcal conjugate vaccine (PCV) introductions in the UK and globally. We previously described concurrent expansion and decline of sequence types associated with serotype replacement over 5 years following PCV introductions in the UK. Here we use whole-genome sequencing to fully characterise the population structure of pneumococcal isolates collected over seven winters encompassing PCV7 and PCV13 introductions in the UK, investigating the importance of lineages in serotype replacement. We analysed 672 pneumococcal genomes from colonised children of 4 years old or less. The temporal prevalence of 20 lineages, defined by hierarchical Bayesian analysis of population structure (BAPS), was assessed in the context of serotype replacement. Multiple serotypes were detected in the primary winter of sampling within three vaccine-type (VT) lineages BAPS4, BAPS10 and BAPS11, in which serotype replacement were observed. In contrast, serotype replacement was not seen in the remaining three VT lineages (BAPS1, BAPS13 and BAPS14), that expressed a single serotype (6B, 6A and 3, respectively) in the primary winter. One lineage, BAPS1 serotype 6B was undetectable in the population towards the end of the study period. The dynamics of serotype replacement, in this UK population, was preceded by the presence or absence of multiple serotypes within VT lineages, in the pre-PCV population. This observation could help predict which non-vaccine types (NVTs) may be involved in replacement in future PCV introductions here and elsewhere. It could further indicate whether any antibiotic resistance associated with the lineages is likely to be affected by replacement.

## Abbreviations

BAPS, Bayesian analysis of population structure; CC, clonal complex; MLST, multi locus sequence typing; NVT, non vaccine serotype; PCV, pneumococcal conjugate vaccine; ST, sequence type (by MLST); VT, vaccine serotype.

## Data Summary

1. All metadata, phylogeny and ENA accession numbers are deposited in microreact: https://microreact.org/project/Southampton-pneumo-carriage.

## Impact Statement

This is the first description of the population structure of this collection of pneumococcal carriage isolates in the UK. This study sought to define dominant lineages and their dynamics over the study period. When a non-vaccine type (NVT) was detected in a vaccine type (VT) lineage in the pre-pneumococcal conjugate vaccine (PCV) era, that NVT was observed to expand, replacing the vaccine serotype. If it can be predicted which NVT/genotype combinations are likely to be involved in replacement, the clinical impact can be assessed, via the lineages antibiotic resistance and virulence determinants profile and the invasive potential of the serotype expressed. Establishing the population structure and serotype mixture of lineages in countries thinking about PCV implementation could therefore offer additional, geographically specific, information during the PCV selection process.

## Introduction

*Streptococcus pneumoniae* has been estimated to be responsible for 5,800 hospitalisations annually in England and Wales before the introductions of pneumococcal conjugative vaccines (PCVs) [1]. Since the introduction of PCV7 in 2006 and PCV13 in 2010, vaccine serotype replacement had nearly completed by winter 2012/13; non-vaccine types account for the majority of carriage pneumococcus isolates from children aged 4 or less, while the prevalence of nasopharyngeal colonisation remained unchanged [2–4]. In contrast, the incidence of invasive disease has fallen since then; disease caused by non-vaccine types (NVTs) did not offset the reduction in vaccine types (VTs) [5, 6].

We previously reported the expansion of sequence types (STs), particularly ST432(21) and ST439(23B), in carriage isolates after the implementation of PCV7; highlighting that clonal expansion played an important role in serotype replacement in the UK [3]. To date, only changes in serotype prevalence have been reported for the additional 2 years post PCV13 implementation [4]. A single vaccine serotype (VT) can be associated with multiple different STs and clonal complexes (CCs), which may influence how replacement will proceed. Therefore, the role of clonal expansions in serotype replacement warranted further investigation. However, previous studies, which only reported multi-locus sequence type (MLST) data (representing <0.2 % of the pneumococcal genome), have limited resolution to fully resolve key relationships within a clone in the pneumococcal population. A clonal complex is defined as STs that share six or more of the seven loci with other STs in the complex, however a single base pair mutation results in a new ST allele. A clonal complex is also limited by the necessity for complete sampling of the connective, single-locus variant, network used to infer relatedness, even in the context of the public databases. Robustly defining lineages beyond ST using higher resolution techniques would allow a detailed analysis of population structure after PCV introductions.

It has been reported that vaccine escape occurring within lineages is a result of selection for existing pneumococci expressing NVT capsules. These variants have been reported to be generated via historical, pre-PCV, capsular switch events [7, 8]. Therefore, we proposed the hypothesis that detection of existing NVT variants in established lineages prior to PCV introduction could be predictive for serotype replacement. Whilst serotypes included in PCVs were selected on the basis of their association with disease, some of the dominant lineages expressing VTs were also associated with high levels of antibiotic resistance. Therefore, assessing indications for lineages being replaced or reduced in the population, could also be predictive of the maintenance through replacement or reduction in antibiotic resistance in these lineages. Additionally the ability of pneumococci to exchange genetic material through homologous recombination varies between lineages and plays a role in both capsular switch variants and the spread of antibiotic resistance [7, 9].

We sought to fully resolve the population structure beyond ST, into discrete lineages for this UK carriage collection. We define the major lineages and relate this to serotype replacement, recombination and prevalence of antibiotic resistance determinants over PCV introductions.

## Methods

Nasopharyngeal swabs were collected from children aged 4 years old or less during seven consecutive winters (October–March) from 2006/7 to 2012/13. The sample collection methodology has been previously reported [3, 10, 11] and approved by UK NHS Research Ethics (06/Q1704/105). Approximately 100 isolates of *S. pneumoniae* were collected each winter. This sample size was powered to detect a 50 % reduction in a 10 % carriage rate at 80 % power with a 5 % type one error rate. Strains were sequenced at The Wellcome Trust Sanger Institute (WTSI) on Illumina HiSeq with Truseq chemistry, paired-end (PE), 75 bp (winters 2006/7–2010/11) and 100 bp (winters 2011/12﻿2012/13).

Reads were mapped against *S. pneumoniae* ATCC 700669 using smalt [12]. The alignment was reduced to variant sites using SNP-sites [13], subjected to hierarchical Bayesian analysis of population structure (hierBAPS) [14] and overlaid on a species-wide RAxML [15] phylogeny to define lineages. Single-nucleotide polymorphisms (SNPs) were reconstructed on the phylogeny using the accelerated transformation Sankoff parsimony method [16]. ariba with the resfinder database was used to detect acquired resistance determinants [17, 18], and penicillin binding protein profiles were determined and used to infer penicillin susceptibility [19] from the fastq files. Lineage-specific references were used to map against for recombination analysis. When a published reference was not available, *de novo* assemblies were produced using a pipeline at WTSI utilising Velvet optimiser [20] and spades [21] and ordered against *S. pneumoniae* ATCC 700669 with ABACAS to provide a draft reference [22] (Table S1). Metrics r/m and rho/theta were calculated for each lineage using Gubbins [23]. The phylogeny, annotated with metadata including accession numbers from the European nucleotide database, was uploaded to permanent microreact URL at: https://microreact.org/project/Southampton-pneumo-carriage. Significant changes in proportions between two groups were detected using Fisher’s exact test with two-tailed *P*-values and adjusted for multiple comparisons using the R p.adjust fdr method. Correlations between measures of recombination and the number of serotypes per lineage, were assessed with the Pearson correlation coefficient. The *P*-value reported was derived from the R score.

## Results

**Fig. 1.**
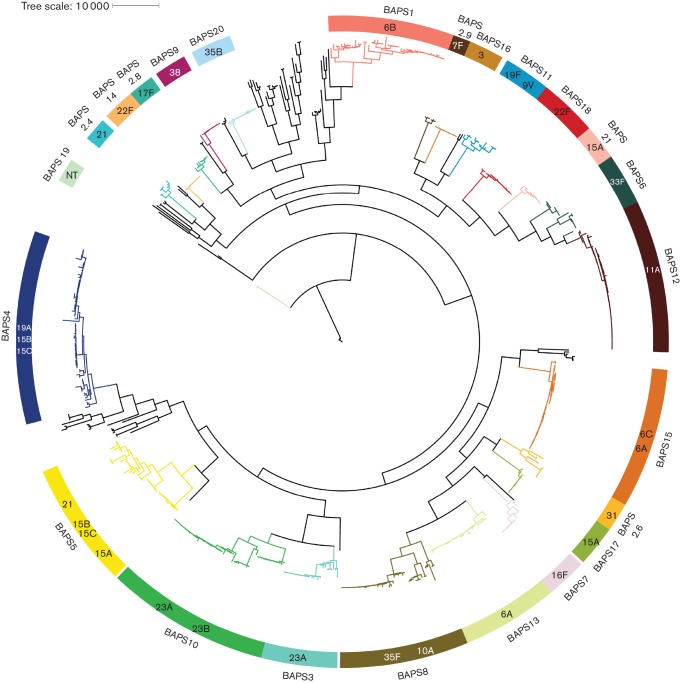
Population structure of the pneumococcal collection, coloured blocks represent each BAPS lineage or sub-lineage of the bin with the corresponding branch coloured accordingly. BAPS lineage blocks are labelled internally with the predominant serotype/s (*n*>2) for the lineage with external labels denoting which BAPS lineage the block refers to.

**Fig. 2.**
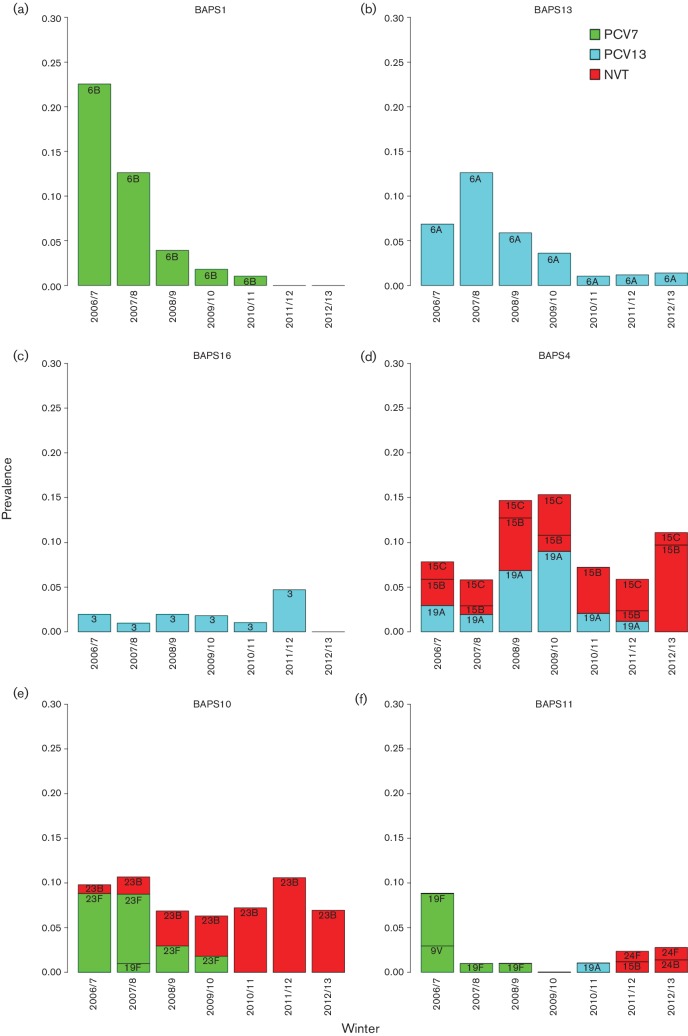
VT lineage temporal serotype dynamics, prevalence of serotypes per VT BAPS lineage each winter. Serotype bars are labelled and coloured based on their respective vaccine inclusion status.

The collection of 672 isolates was clustered into 20 BAPS lineages and a polyphyletic bin by the primary hierarchical clustering of hierBAPS [14] (Fig. 1). Three lineages (BAPS 1, 10 and 11) were dominated by PCV7 VTs and a further three (BAPS 4, 13 and 16) by PCV13 VTs together comprising >50 % of the isolates collected in the primary winter of this study, referred to here as the starting population (Fig. 2).

### Lineage extinction

The CCs and STs described here are encompassed by the BAPS lineages, but not equivalent to the broader designations of BAPS. Lineage BAPS1, which encompasses ST176, exclusively expressed PCV7 vaccine serotype 6B, and decreased in prevalence significantly between the primary winter 2006/7 and post-PCV7 winter 2009/10 before PCV13 introduction (*P*<0.0001) after adjusting for multiple testing (Fig. 2a). The striking decrease in this lineage culminated in its absence in the final two winters (2011/12 and 2012/13) of this study, with no resurgence of 6B or the STs encompassed by this lineage in winters 2013/14–2015/16 subsequent to 2012/13 in this ongoing carriage surveillance study (our unpublished data).

BAPS13, which includes ST65, exclusively expressed PCV13 serotype 6A. BAPS13(6A) increased temporarily after the introduction of PCV7 but decreased soon after the introduction of PCV13. In 2012/13 only one 6A isolate was detected in this study, and no serotype replacement was observed for BAPS13(6A) (Fig. 2b). Furthermore, serotype 6A was absent from the carriage study population in subsequent winters of 2013/14 and 2015/16 (unpublished data).

Serotypes 6B and 6A were two of the top three serotypes reported in the primary winter 2006/7 [10]. The majority of which were encompassed by the two lineages BAPS1(6B) and BAPS13(6A). These two lineages showed the largest decreases during the study and both lineages exclusively expressed their respective serotypes 6B and 6A. BAPS16 [3], dominated by ST180, exclusively expressed PCV13 VT 3 (Fig. 2c) and also appears to have been nearly removed from the population, with only one serotype 3 (ST180) detected between the subsequent winters 2013/14 and 2015/16 (unpublished data). Significant reductions were not detected for BAPS13 and BAPS16 (Table S1, available in the online Supplementary Material), although replacement may not be complete only 3 years after the introduction of PCV13 in 2010 [24].

### Lineage serotype replacement

The remaining three VT lineages, BAPS4, BAPS10 and BAPS11, all exhibited serotype replacement, and thus were maintained in the population. BAPS4, which encompasses ST199, transitioned from a mixed population of PCV13 VT 19A and NVT 15B/C, to exclusively 15B/C over the seven winters (Fig. 2d). BAPS10 was initially dominated by PCV7 VT 23F and predominantly ST36. Serotype 23F was completely replaced by 23B, which was predominantly ST439 (Fig. 2e). In both these instances the replacing serotype was present in the starting population for that lineage and multiple STs were involved that previously obscured the replacement occurring at the lineage level as determined using only MLST data [3]. The replacing serotypes 15B/C and 23B were among the top five serotypes by winter 2012/13.

Members of BAPS11 belonging to ST162 initially expressed VTs 19F and 9V, and decreased significantly after the introduction of PCV7 (*P*=0.02). However, BAPS11 continued to persist in the population at a low level with 19F replaced by multiple different serotypes through the study period including serogroup 24 [24F (*n*=2), 24B (*n*=1)] (Fig. 2f). Serogroup 24 isolates were not observed within BAPS11 in the primary winter 2006/7. However, the phylogeny indicates that the BAPS11 serogroup 24 isolates shared a most recent common ancestor with all 19F strains including those 19Fs present in the starting population. This lineage, specifically ST162, went on to cause four disease cases post-PCV13 in the Hampshire region, expressing serotype 24F. The remaining PCV VTs were detected in small numbers during the study and do not play a major role in the dynamics of this population structure.

### Lineage expansion

Changes in prevalence of NVT lineages BAPS12(11A, CC62) and BAPS17(15A, CC58) during the study were not statistically significant (Fig. 1 and Table S1). Significant increases between winter 2006/7 and 2009/10 for BAPS5(21, CC193) and BAPS18(22F, CC433) were non-significant after adjusting for multiple testing. No significant increase was observed between 2006/7 and 2012/12 for these two lineages. Individual NVT lineages exhibiting serotype exclusivity in the pre-PCV period did not expand significantly after PCV introductions, with no single NVT lineage offsetting the striking removal of VT lineages like BAPS1(6B) in this sampling.

### Antibiotic resistance

Low prevalence and sporadic acquisition of antibiotic resistance determinants were detected in this population. As only limited antibiotic phenotype data was available for this dataset, resistance was inferred from the presence of known genetic determinants of resistance. Work published by Metcalf *et al.* allowed us to make inferences of penicillin susceptibility from the penicillin binding protein allele profiles [25]. The profiles were used to predict that the isolates have minimum inhibitory concentrations of <0.03 µg ml^−1^, and can be classed as susceptible to penicillin [19, 25, 26]. Only 16 % of isolates had acquired non-core genes known to confer resistance, 5.5 % of isolates had acquired more than one resistance gene, and only 1 % had acquired more than two. The acquired resistance genes detected, including *mef*, *msr*D, *tet*M and *erm*B, are typical for the pneumococcal species, though their prevalence in this population is low [27]. A phylogenetic cluster of acquired resistance genes, could be observed for BAPS13(6A), that had acquired the *mefE* gene for macrolide resistance. Whilst this lineage decreased in prevalence during the study, BAPS8(35F/10A), also containing *mefE*, temporarily increased and so maintained the gene within the population. The increase in BAPS8 was not sustained through the study period, with a non-significant difference between 2006/7 and 2012/13. BAPS13(6A, CC65) and BAPS8(35F/10A, CC1635) share a common ancestor before they share one with any other BAPS lineage, indicating that their common ancestor was likely to be *mefE*-positive. NVT BAPS21(15A) carried *tet*M and *erm*B but its prevalence never exceeded 0.05 in any given year and a significant increase was not detected within the study period. There was no clonal acquired resistance associated with the lineages being removed from the population, or differences between the VT and NVT components in lineages that were replaced, leaving the resistance profile of the lineages and the population unchanged.

### Recombination

Both r/m and rho/theta vary between lineages, in this population BAPS lineages with a high recombination to mutation ratio (r/m), also tended to have a high number of recombination events to point mutations ratio (rho/theta) (Table S1). For the majority of lineages r/m was greater than one and thus recombination introduced more genetic variation than point mutations. A moderate positive correlation was observed between the number of recombination events and number of serotypes for each BAPS lineage R=0.534, *P*=0.01525 in concordance with the correlation reported by Croucher *et al.* [9].

## Discussion

We observed that VT lineages expressing multiple serotypes in the starting population underwent serotype replacement and were retained in the carriage population after PCV introductions. Conversely lineages expressing exclusively VT serotypes decreased to almost undetectable levels at the end of the study period. Pneumococcal sequencing has been employed by numerous research groups to gain further insight into its biology [28–33]. Only three other studies, however, have assessed the species-wide population structure in the context of PCVs [7, 34, 35]. To determine if our findings in this carriage collection could be extrapolated to other geographical locations we made a comparison with the one other study that describes changes in prevalence of BAPS clusters pre and post PCV7 [7]. The UK collection reported here has numerous observable similarities to the pneumococcal carriage population from Massachusetts [7]. The collections have similar numbers of lineages defined by hierBAPS and include considerable polyphyletic bins of low-frequency genotypes accounting for 17 % (UK) and 20 % (Massachusetts). There is considerable overlap when using STs and CCs as a proxy for comparing BAPS lineages in the two populations. This approach established which BAPS lineages were shared for the subsequent comparison.

Four BAPS clusters in Massachusetts expressed PCV7 VTs in 2001. All four underwent replacement to varying extents. In Massachusetts BAPS9 (23F), known as sequence cluster (SC) 9 in the original paper, replacement was observed that could be expected with our hypothesis given the presence of 23A and 23B in 2001. This mirrored the replacement of BAPS10(23F), its counterpart in the UK, that encompassed ST439 and ST3636. Unlike the UK collection, the remaining three PCV7 VT lineages in Massachusetts were replaced by NVTs that were not detected in the 2001 sampling. The Massachusett VT lineages were a much smaller proportion of the starting population the in the UK (17 and 57 % respectively). It is likely that the NVTs were present in the population but went unsampled in 2001 [7].

Sample size is critical to properly assess diversity within a population and to detect changes. A sufficient sample with enough statistical power can reduce the likelihood of any minority serotypes/lineages going undetected. Any sampling designed to establish the population structure could additionally be complemented with data on serotype and STs from pubMLST. For example, an isolate from Italy, of ST162 24F observed in 1998, is reported in the public MLST database, further indicating that the BAPS11 24F capsular variant that replaced 19F was generated through capsular switching pre-PCV and circulating in Europe. Additionally, BAPS11 did express two unrelated PCV7 VTs (19F, 9V) in 2006/7 from a sample of nine strains. This may indicate that BAPS11 has a propensity for capsule switching and serogroup 24 may have been uncovered with deeper sampling. Screening the MLST databases would determine, for a given lineage, whether any unsampled NVTs have previously been documented in the same location and time frame. Although pubMLST may have a bias towards rare and novel serotype ST combinations. It is possible that a low-frequency serotype mixture is present in all lineages and that serotype exclusivity would not be observed with exhaustive sampling. Constraints on serotype switching is an area of ongoing investigation which may add insight into whether capsule switches occur in all lineages [9]. However, the presence of NVTs in the starting population, even in a small number of samples, appears to be a strong indication for subsequent replacement within that lineage. For resource-limited countries where PCVs are still being introduced via national immunization programs, preliminary studies on the pre-PCV population structures have been published [30, 35, 36]. When post-PCV data is available it will be informative to compare these to the early PCV adopters in the resource-rich countries of Europe and North America, especially given the different burden of disease and serotype prevalence [37].

In addition to the patterns of replacement described, similar serotype mixes were observed for some lineages in both Massachusetts and our UK collection. Massachusetts lineage SC8 and its equivalent UK BAPS4 are characterised by CC199. Both locations included 19A and 15B/C at near equal proportions in these lineages in the initial sampling. After PCV13, replacement of 19A by 15B/C in this lineage was observed in both populations despite the geographical distance [38]. Subsequent increases in 15B/C IPD cases were observed in the UK and USA [6, 39]. The presence of 19A and 15B/C within the same STs and CCs is also observable in the pneumococcal pubMLST database involving multiple lineages globally [40]. This indicates that the replacement of 19A by 15B/C post-PCV could also be anticipated for other countries adopting PCVs. For this lineage 15B/C has also been shown to be as equally capable as 19A of causing middle ear infections in chinchilla [41]. Therefore, 15B/C may be a potential target for inclusion in future PCVs.

Serotype replacement has been reported across the world [3, 5, 24, 34, 42–46]. Certain serotypes have increased post-PCV in multiple locations, globally, such as 19A post-PCV7 for disease and carriage [8, 43, 47–49]. However, we and others have shown that serotype replacement involves clonal lineages [3, 44, 50–52], therefore it is useful to consider genotype alongside serotype to understand the replacement. The extent that different serotypes are involved in replacement can differ between countries, and is likely to be directly linked to the prevalence of certain genotypes in the starting population, with each location having a unique population structure. For example, the Massachusetts lineage SC1(6A) relates to BAPS13(6A). In Massachusetts however, SC1 expressed multiple serotypes in the starting population. This demonstrates that the pattern of replacement for a given lineage in one country could not predict whether that replacement will occur for the same lineage in another. Serotype replacement will be specific to the starting population in both temporal and spatial terms and reservoirs at the boundaries of the geographic population, for example, differing use of PCVs in neighbouring countries may also need to be considered.

Lineage-specific antibiotic resistance profiles in this UK collection were detected in dominant lineages BAPS8, 13 and 21, potentially contributing to the success of these lineages. However, with only low-level prevalence of antimicrobial resistance, it appears not to be essential for the success of carriage lineages in the UK. While there were reports of rises in antibiotic resistance in NVTs causing disease after the introduction of PCV7 in the USA, there was no evidence of any rise in this UK population [27].

It is of interest to consider both the resistance profile and serotype makeup of a NVT component of a pre-PCV lineage with potential to be involved in replacement. In this case resistance was near absent within the NVTs involved in replacement and the invasiveness of the NVT capsule, measured by odds ratios [53, 54], was similar to that of the VTs they were replacing. It would be of concern if a NVT component of a VT lineage had a capsule known to be more invasive than the VT it could replace, influencing the rate at which the lineage could translate into disease cases. Furthermore, if the NVT component of a pre-PCV lineage contained fewer resistance determinants than its VT counterpart, even with the prevalence of the lineage remaining stable it could, in theory, reduce the contribution of that lineage to resistance in that population and influence the ability to treat pneumococcal infections from that lineage.

Genomic surveillance of carriage increasingly has the advantage of being rapidly deployed for the capture of the dynamics of pneumococcal population structure over PCV introductions. A combination of lineage extinction, replacement and expansion of multiple low-frequency genotypes will contribute to post-PCVs population structure. These processes combined have resulted in the stable carriage prevalence observed for this collection over time [3, 4, 10]. Genomic population datasets such as this also lend themselves to modelling the impact of PCVs on a given population, which may offer greater predictive value for assessing implementations of PCVs.

### Conclusion

Here we give the first description, of the population structure for this UK carriage collection. This allowed us to observe that serotype exclusivity within a lineage during the primary sampling winter was an indicator that the lineage would go on to be removed from circulation rather than undergo replacement with unsampled NVTs. Conversely the presence of multiple serotypes in the starting population of a VT lineage indicated that the lineage would undergo serotype replacement following PCV. When a NVT was sampled in the primary winter this NVT went on to be the replacing type in this study. There were no significant expansions of individual NVT lineages in this sampling. Observed significant increases in NVTs post-PCV are as a result of the combined NVT replacement through expansion of multiple lineages. As serotype replacement is influenced by the genotypic background, identifying VT lineages at risk of serotype replacement could additionally assess the added impact of the antibiotic resistance profile or virulence determinants such as capsule of the NVT component. These observations could be used to inform PCV selection or future design of extended valency PCVs for a given population.

## Supplementary Data

43 Supplementary File 1

## Supplementary Data

43 Supplementary File 2

## References

[1] Melegaro A, Edmunds WJ, Pebody R, Miller E, George R (2006). The current burden of pneumococcal disease in England and Wales. J Infect.

[2] Ladhani SN, Slack MP, Andrews NJ, Waight PA, Borrow R (2013). Invasive pneumococcal disease after routine pneumococcal conjugate vaccination in children, England and Wales. Emerg Infect Dis.

[3] Gladstone RA, Jefferies JM, Tocheva AS, Beard KR, Garley D (2015). Five winters of pneumococcal serotype replacement in UK carriage following PCV introduction. Vaccine.

[4] Devine VT, Cleary DW, Jefferies JM, Anderson R, Morris DE (2017). The rise and fall of pneumococcal serotypes carried in the PCV era. Vaccine.

[5] Miller E, Andrews NJ, Waight PA, Slack MP, George RC (2011). Herd immunity and serotype replacement 4 years after seven-valent pneumococcal conjugate vaccination in England and Wales: an observational cohort study. Lancet Infect Dis.

[6] Waight PA, Andrews NJ, Ladhani NJ, Sheppard CL, Slack MP (2015). Effect of the 13-valent pneumococcal conjugate vaccine on invasive pneumococcal disease in England and Wales 4 years after its introduction: an observational cohort study. Lancet Infect Dis.

[7] Croucher NJ, Finkelstein JA, Pelton SI, Mitchell PK, Lee GM (2013). Population genomics of post-vaccine changes in pneumococcal epidemiology. Nat Genet.

[8] Brueggemann AB, Pai R, Crook DW, Beall B (2007). Vaccine escape recombinants emerge after pneumococcal vaccination in the United States. PLoS Pathog.

[9] Croucher NJ, Kagedan L, Thompson CM, Parkhill J, Bentley SD (2015). Selective and genetic constraints on pneumococcal serotype switching. PLoS Genet.

[10] Tocheva AS, Jefferies JM, Rubery H, Bennett J, Afimeke G (2011). Declining serotype coverage of new pneumococcal conjugate vaccines relating to the carriage of *Streptococcus pneumoniae* in young children. Vaccine.

[11] Tocheva AS, Jefferies JM, Christodoulides M, Faust SN, Clarke SC (2013). Distribution of carried pneumococcal clones in UK children following the introduction of the 7-valent pneumococcal conjugate vaccine: a 3-year cross-sectional population based analysis. Vaccine.

[12] Wellcome Trust Sanger Institute (2014). SMALT. www.sanger.ac.uk/science/tools/smalt-0.

[13] Page AJ, Taylor B, Delaney AJ, Soares J, Seemann T (2016). SNP-sites: rapid efficient extraction of SNPs from multi-FASTA alignments. Microb Genom.

[14] Cheng L, Connor TR, Sirén J, Aanensen DM, Corander J (2013). Hierarchical and spatially explicit clustering of DNA sequences with BAPS software. Mol Biol Evol.

[15] Stamatakis A (2006). RAxML-VI-HPC: maximum likelihood-based phylogenetic analyses with thousands of taxa and mixed models. Bioinformatics.

[16] Sankoff D (1975). Minimal mutation trees of sequences. SIAM J Appl Math.

[17] Wellcome Trust Sanger Institute (2016). ARIBA. https://github.com/sanger-pathogens/ariba/wiki.

[18] Zankari E, Hasman H, Cosentino S, Vestergaard M, Rasmussen S (2012). Identification of acquired antimicrobial resistance genes. J Antimicrob Chemother.

[19] Metcalf BJ, Gertz RE, Gladstone RA, Walker H, Sherwood LK (2016). Strain features and distributions in pneumococci from children with invasive disease before and after 13-valent conjugate vaccine implementation in the USA. Clin Microbiol Infect.

[20] Victorian Bioinformatics Consortium (2008). Velvet optimiser. https://github.com/Victorian-Bioinformatics-Consortium/VelvetOptimiser.

[21] Bankevich A, Nurk S, Antipov D, Gurevich AA, Dvorkin M (2012). SPAdes: a new genome assembly algorithm and its applications to single-cell sequencing. J Comput Biol.

[22] Wellcome Trust Sanger Institute (2015). ABACAS. http://abacas.sourceforge.net/.

[23] Croucher NJ, Page AJ, Connor TR, Delaney AJ, Keane JA (2015). Rapid phylogenetic analysis of large samples of recombinant bacterial whole genome sequences using Gubbins. Nucleic Acids Res.

[24] Hanage WP, Finkelstein JA, Huang SS, Pelton SI, Stevenson AE (2010). Evidence that pneumococcal serotype replacement in Massachusetts following conjugate vaccination is now complete. Epidemics.

[25] Metcalf BJ, Chochua S, Gertz RE, Li Z, Walker H (2016). Using whole genome sequencing to identify resistance determinants and predict antimicrobial resistance phenotypes for year 2015 invasive pneumococcal disease isolates recovered in the United States. Clin Microbiol Infect.

[26] Li Y, Metcalf BJ, Chochua S, Li Z, Gertz RE (2016). Penicillin-binding protein transpeptidase signatures for tracking and predicting β-lactam resistance levels in *Streptococcus pneumoniae*. MBio.

[27] Farrell DJ, Klugman KP, Pichichero M (2007). Increased antimicrobial resistance among nonvaccine serotypes of *Streptococcus pneumoniae* in the pediatric population after the introduction of 7-valent pneumococcal vaccine in the United States. Pediatr Infect Dis J.

[28] Cleary DW, Devine VT, Jefferies JM, Webb JS, Bentley SD (2016). Comparative genomics of carriage and disease isolates of *Streptococcus pneumoniae* serotype 22F reveals lineage-specific divergence and niche adaptation. Genome Biol Evol.

[29] Deng X, Peirano G, Schillberg E, Mazzulli T, Gray-Owen SD (2016). Whole-genome sequencing reveals the origin and rapid evolution of an emerging outbreak strain of *Streptococcus pneumoniae* 12F. Clin Infect Dis.

[30] Chewapreecha C, Harris SR, Croucher NJ, Turner C, Marttinen P (2014). Dense genomic sampling identifies highways of pneumococcal recombination. Nat Genet.

[31] Roca A, Bojang A, Bottomley C, Gladstone RA, Adetifa JU (2015). Effect on nasopharyngeal pneumococcal carriage of replacing PCV7 with PCV13 in the Expanded Programme of Immunization in The Gambia. Vaccine.

[32] Loman NJ, Gladstone RA, Constantinidou C, Tocheva AS, Jefferies JM (2013). Clonal expansion within pneumococcal serotype 6C after use of seven-valent vaccine. PLoS One.

[33] Gladstone RA, Gritzfeld JF, Coupland P, Gordon SB, Bentley SD (2015). Genetic stability of pneumococcal isolates during 35 days of human experimental carriage. Vaccine.

[34] Cremers AJ, Mobegi FM, de Jonge MI, van Hijum SA, Meis JF (2015). The post-vaccine microevolution of invasive *Streptococcus pneumoniae*. Sci Rep.

[35] Everett DB, Cornick J, Denis B, Chewapreecha C, Croucher N (2012). Genetic characterisation of Malawian pneumococci prior to the roll-out of the PCV13 vaccine using a high-throughput whole genome sequencing approach. PLoS One.

[36] Brueggemann AB, Muroki BM, Kulohoma BW, Karani A, Wanjiru E (2013). Population genetic structure of *Streptococcus pneumoniae* in Kilifi, Kenya, prior to the introduction of pneumococcal conjugate vaccine. PLoS One.

[37] Hausdorff WP, Bryant J, Paradiso PR, Siber GR (2000). Which pneumococcal serogroups cause the most invasive disease: implications for conjugate vaccine formulation and use, part I. Clin Infect Dis.

[38] Lee GM, Kleinman K, Pelton SI, Hanage W, Huang SS (2014). Impact of 13-valent pneumococcal conjugate vaccination on *Streptococcus pneumoniae* carriage in young children in Massachusetts. J Pediatric Infect Dis Soc.

[39] Makarewicz O, Lucas M, Brandt C, Herrmann L, Albersmeier A (2017). Whole genome sequencing of 39 invasive *Streptococcus pneumoniae* sequence type 199 isolates revealed switches from serotype 19A to 15B. PLoS One.

[40] MLST (2014). *Streptococcus pneumoniae* MLST Database: University of Oxford. pubMLST. http://pubmlst.org/spneumoniae.

[41] Laufer AS, Thomas JC, Figueira M, Gent JF, Pelton SI (2010). Capacity of serotype 19A and 15B/C *Streptococcus pneumoniae* isolates for experimental otitis media: implications for the conjugate vaccine. Vaccine.

[42] Rodrigues F, Foster D, Caramelo F, Serranho P, Gonçalves G (2012). Progressive changes in pneumococcal carriage in children attending daycare in Portugal after 6 years of gradual conjugate vaccine introduction show falls in most residual vaccine serotypes but no net replacement or trends in diversity. Vaccine.

[43] Carnalla-Barajas MN, Soto-Noguerón A, Sánchez-Alemán MA, Solórzano-Santos F, Velazquez-Meza ME (2017). Changing trends in serotypes of *S. pneumoniae* isolates causing invasive and non-invasive diseases in unvaccinated population in Mexico (2000-2014). Int J Infect Dis.

[44] Ardanuy C, Tubau F, Pallares R, Calatayud L, Domínguez MA (2009). Epidemiology of invasive pneumococcal disease among adult patients in barcelona before and after pediatric 7-valent pneumococcal conjugate vaccine introduction, 1997–2007. Clin Infect Dis.

[45] Hernandez-Bou S, Garcia-Garcia JJ, Gene A, Esteva C, del Amo E (2012). Pneumococcal carriage in children attending a hospital outpatient clinic in the era of pneumococcal conjugate vaccines in Barcelona. Diagn Microbiol Infect Dis.

[46] Plessis MD, Ld G, Allam M, Ndlangisa K, Wolter N Non-vaccine pneumococcal serotypes in adults aged ≥25 years pre and post pneumococcal conjugate vaccine introduction in South Africa. Proceedings of the 10th International Symposium on Pneumococci & Pneumococcal Disease.

[47] Pan F, Han L, Kong J, Wang C, Qin H (2015). Serotype distribution and antimicrobial resistance of *Streptococcus pneumoniae* causing noninvasive diseases in a Children's Hospital, Shanghai. Braz J Infect Dis.

[48] van der Linden M, Falkenhorst G, Perniciaro S, Imöhl M (2015). Effects of infant pneumococcal conjugate vaccination on serotype distribution in invasive pneumococcal disease among children and adults in Germany. PLoS One.

[49] Spijkerman J, van Gils EJ, Veenhoven RH, Hak E, Yzerman EP (2011). Carriage of *Streptococcus pneumoniae* 3 years after start of vaccination program, the Netherlands. Emerg Infect Dis.

[50] Elberse KE, Wagenvoort GH, Pluister GN, de Melker HE, Sanders EA (2016). Pneumococcal population in the era of vaccination: changes in composition and the relation to clinical outcomes. Future Microbiol.

[51] Slotved HC, Dalby T, Hoffmann S (2015). Multilocus sequence types of invasive pneumococcal isolates from Danish infants (0–90 days) 2003–2013. BMC Res Notes.

[52] Antonio M, Dada-Adegbola H, Biney E, Awine T, O'Callaghan J (2008). Molecular epidemiology of pneumococci obtained from Gambian children aged 2–29 months with invasive pneumococcal disease during a trial of a 9-valent pneumococcal conjugate vaccine. BMC Infect Dis.

[53] Brueggemann AB, Griffiths DT, Meats E, Peto T, Crook DW (2003). Clonal relationships between invasive and carriage *Streptococcus pneumoniae* and serotype- and clone-specific differences in invasive disease potential. J Infect Dis.

[54] Brueggemann AB, Peto TE, Crook DW, Butler JC, Kristinsson KG (2004). Temporal and geographic stability of the serogroup-specific invasive disease potential of *Streptococcus pneumoniae* in children. J Infect Dis.

