# Supplementary figures and images for: Pre-vaccine serotype composition within a lineage signposts its serotype replacement – a carriage study over 7 years following pneumococcal conjugate vaccine use in the UK
Source: Microb Genom. 2017 Jun 9;3(6):e000119. doi: 10.1099/mgen.0.000119 (PMC5628697; doi:10.1099/mgen.0.000119)

Supplementary Figure 1. BAPS cluster prevalence over study period

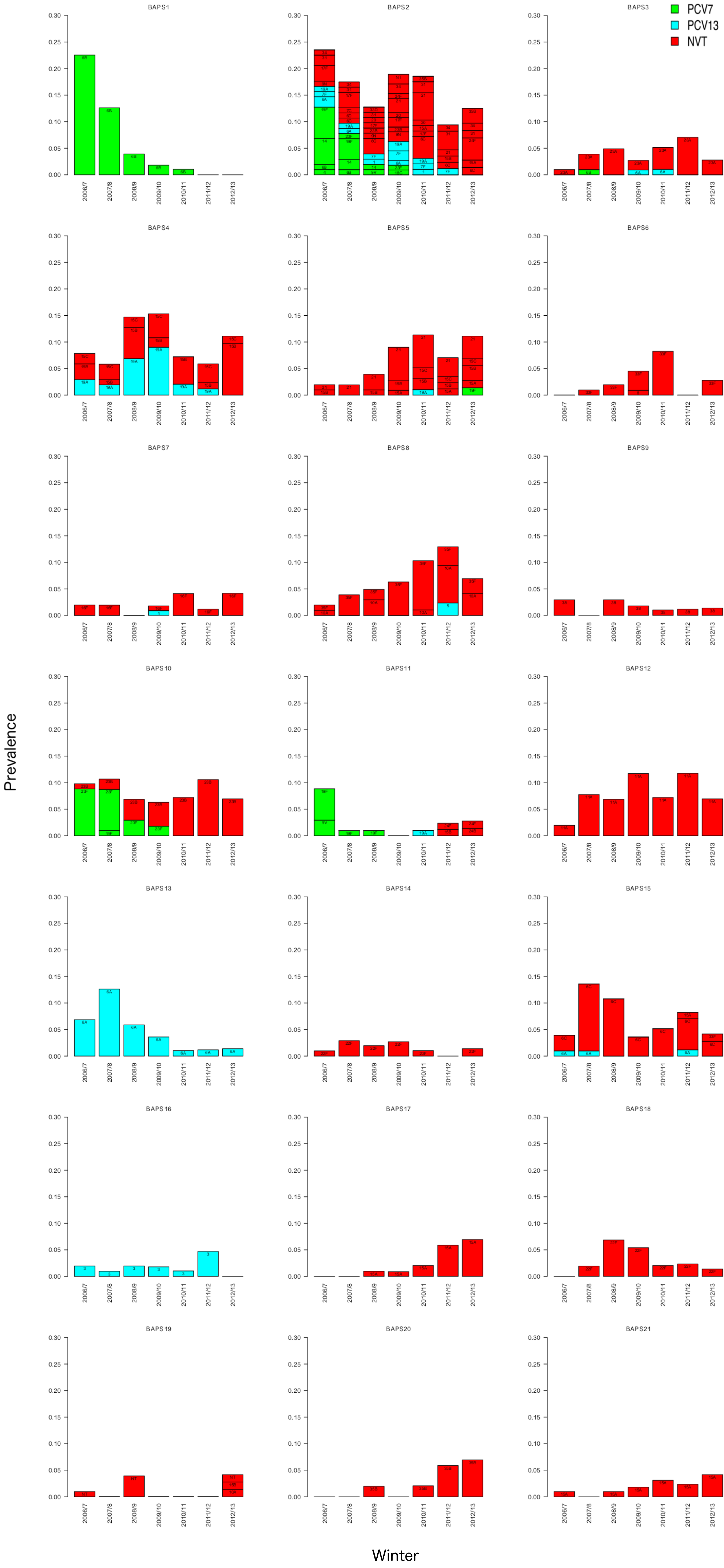

Supplement: Supplementary File 2 [file mgen-3-119-s002.pdf]
